# Book Reviews

**Published:** 1876-12

**Authors:** 


### Book Reviews.

[NOTE.—All works reviewed in the pages of the CHICAGO MEDICAL JOURNAL AND EXAMINER may be found in the extensive stock of W. B. KEEN, COOKE & Co., whose Catalogue of Medical Books will be sent to any address upon request.]

CLINICAL LECTURES ON DISEASES OF THE HEART AND AORTA, by *George William Balfour, M.D., St.And. F.R.C.P. Ed.*, Physician to, and Lecturer on Clinical Medicine in the Royal Infirmary, Edinburgh. Philadelphia: Lindsay & Blakiston. 1876.

This is a work of 424 pages, relating to the diagnosis and treatment of cardiac diseases and affections of the aorta. It has the form of a series of clinical lectures; not that any such lectures were really delivered, but because the author found it more convenient to express himself in this manner.

The book is written in that positive style—*never sustained by experience*—which is so attractive to students and a class of medical men who are constantly searching for specifics in therapeutics. This style will add to its popularity with many, and the qualification of some of the more positive statements will give it the confidence of thoughtful readers.

Upon the first page we find this comforting statement for nervous and dyspeptic individuals who suppose themselves the victims of incurable disease of the heart: "Should a patient come to you complaining of palpitation, irregular action, or of his heart generally, you may at once assure him, without much fear of being wrong, that his heart is all right."

We are taught the difference between pulmonary and cardiac breathlessness, and shown how the latter may be dependent simply upon blood changes.

The character of the arterial pulse, in various forms of cardiac disease, is described; and we are told that visible venous pulsation is invariably a sign of considerable dilatation of the right side of the heart. However, we must not suppose that this dilatation is necessarily due to organic disease of the heart, for it may have no other cause than obstruction to the flow of blood through the lungs, resulting perhaps from simple bronchitis.

The author considers superficial dullness of no importance in determining the size of the heart. He recommends that deep-seated dullness be determined from above downward, on a vertical line one inch to the left of the sternum, and that the extent of lateral dullness be ascertained on the plane of the fourth rib. We are reminded that the apex beat may vary an inch or more from its ordinary location, with changes in the position of the patient.

An accentuated, pulmonary second sound is said to be the most persistent of all the acoustic phenomena indicative of cardiac disease, and it is frequently the *only* distinctly abnormal sound to be detected. The author holds that accentuation of this sound, if unconnected with any pulmonary disease capable of producing congestion of the lungs, invariably indicates valvular lesions, and accentuation of the aortic second sound is invariably a sign of dilatation of the aorta or arteria innominata.

Regarding reduplication of the heart sounds, he thinks the first sound becomes reduplicated, because excess of blood pressure in the heart retards the closure of the tricuspid valves; and the second sound is reduplicated because excess of pressure in the aorta accelerates the occlusion of the aortic valves.

Murmurs are thought to be of little value in diagnosticating disease of the heart. He says: "It is obvious that murmurs cannot of themselves be accepted as certain indications of cardiac disease, even although we can positively connect them with the lesion of a definite valve, because that lesion may be temporary in its character, and wholly unconnected with actual disease of the valve affected. To this there is but one positive exception — the so-called presystolic murmur." The latter murmur — indicative of mitral stenosis — is usually thought to be very uncommon; yet we are told that it is one of the most common of endocardial murmurs, and the one most easily detected. The presystolic — or, as the author terms it, the auriculo-systolic-murmur — is described as a rough sound capable of being represented phonetically by the symbols

*Rrrb* or *Voot*, a sound preceding the apex beat and carotid pulse, and usually running quite up to them; separated from the second sound of the heart by an appreciable interval, and almost invariably accompanied by a purring tremor over the mitral area. He does not enter into a discussion of the manner in which this sound is produced, but simply states his views — cites some of the objections, and refers the reader who cares to pursue the subject to an article published in the *Lancet*, May 25th, 1872, p. 714.

The causes for the irregular transmission of aortic murmurs are not well understood; therefore, we are pleased to find in this work a very plausible explanation of the matter. This explanation would lead us to believe it easy to diagnose the exact condition of the aortic valves by the quality of the murmur and its area of diffusion.

The quality of an endocardial murmur is generally thought to be the least in importance of its elements; but we are told that this element alone is often sufficient to establish the diagnosis.

Regarding aortic diastolic murmurs, we find the following interesting summary:

"A diastolic murmur at or below the level of the aortic valves, chiefly audible in the line of the sternum, is significant of aortic incompetence. If this diastolic murmur be inaudible in the carotid arteries, it is invariably accompanied by a systolic murmur, having its maximum intensity at the aortic valves, or in the so-called aortic area; and this indicates comparatively trifling incompetence, with considerable obstruction at the aortic orifice, most probably from calcified semilunar valves. If this diastolic murmur be distinctly audible in the carotid arteries, it is invariably preceded by a loud systolic murmur in them, the systolic portion of the murmur being not always very audible in the aortic or in any part of the cardiac area; and this indicates very considerable incompetence, with comparatively trifling obstruction."

The first effect of aortic regurgitation is to overload the left ventricle, a condition which gives rise to various distressing symptoms — pain, palpitation, etc. We are told that to overcome this condition the left ventricle dilates, so as to make room for the increased amount of blood; but as dilatation would only relieve the heart temporarily, the ventricular walls become hypertrophied.

Many writers believe that hypertrophy may pass beyond the degree sufficient to overcome the obstruction to the circulation, and thus become an element of danger. We believe the author right in thinking this state of matters extremely rare, and in even doubting its possibility. He thinks the prognosis more unfavorable in aortic incompetence than in a similar condition of the mitral valves, and that in the former, sudden death is more frequent, so "That the probability of life is very greatly in favor of mitral lesions."

In the treatment of aortic incompetence reliance is placed principally upon Digitalis, in doses of m. xv. to ʒ ss. of the tincture, every four hours. Smaller doses are sometimes useful; but these larger doses are usually needed, and they are considered perfectly safe if watched. When giving these large doses, we should at once suspend the use of the medicine if the quantity of urine diminishes, or at least as soon as the pulse begins to falter or nausea supervenes; but the same precaution is not necessary with small doses, and we need not fear evil effects from the large doses unless these symptoms occur.

For the neuralgic pain which so often accompanies this disease, arsenic is recommended. "In all forms of cardiac angina arsenic is almost a specific, and in this form it certainly acts with great benefit."

We look with suspicion upon medicines which are recommended as "almost specifics;" yet there can be no harm in trying the arsenic, for it is thought to strengthen the cardiac walls.

"A systolic apex murmur is by no means always a certain proof of any positive derangement of the cardiac mechanism." "Even though regurgitation be unequivocally proved to exist, along with a systolic apex bruit, mitral disease or deformity is not therefore a necessary consequent, because both do occur in a not inconsiderable proportion of cases in which the mitral valve is nevertheless perfectly healthy." To many this statement may appear singular, yet we think it will be found correct; for there are numerous cases in which the heart, weakened by disease, dilated, so that the auriculo-ventricular openings become too large to be closed by the valves. With regard to this the author says that cardiac dilatation is invariably revealed by mitral regurgitation, which, in more severe forms, is associated with tricuspid regurgitation also; and this dilatation may occur independently of valvular disease, giving rise to curable mitral or tricuspid regurgitation.

In curable mitral regurgitation, it is common to obtain a murmur over the left auricle, synchronous with the systole of the ventricles. This murmur is usually most distinct about an inch and a half to the left of the pulmonary area. It can generally be heard over the aorta and carotids (when it is usually termed an anæmic murmur); in some cases it may also be heard at the apex.

Among the diseases which may give rise to curable mitral regurgitation, the author enumerates typhus, enteric and relapsing fevers; erysipelas, chorea, and some forms of anæmia; and he thinks it probable many others may have a similar effect. Curable mitral regurgitation is to be treated by general tonics, digitalis and arsenic.

The frequent disappearance of endocardial murmurs in organic disease, as well as their occurrence where no actual cardiac disease exists, renders it important for us to be able to diagnose valvular lesions independently of murmurs; and, fortunately, this is not very difficult, as the author has shown in a case illustrative of the manner in which we are to proceed by the process of exclusion.

One chapter is devoted to the subject of irregularities of the pulse, and arterial and cardiac palpitation; and another, of special interest, to the secondary results of cardiac disease.

In an article on angina pectoris, chloroform is said to be the best remedy during the attack; but æther, nitrite of amyl and morphia are also recommended. In this connection we find the following statement regarding the use of chloroform:

"I know of no diseased condition which should deter us from its cautious employment where that is otherwise indicated, as I hold it imperatively to be in cases of angina pectoris; for what we desire to do in such cases can only be done by means of chloroform."

In this the author refers to the speedy relief of pain. He recommends giving the patient a small chloroform smelling-bottle, the fluid being poured over a sponge, so that it cannot spill. This the patient holds to the nose, breathing deeply as possible until relief is obtained. When the narcotic influence is obtained the bottle drops from the patient's hand, and "with it rolls away all risk of an overdose."

The author doubts the possibility of diagnosing a fatty heart, though he says we may have strong reasons for suspecting it, and our suspicions may often be correct.

The last chapter is devoted to the treatment of internal aneurism by iodide of potassium. Referring to other plans of treatment, the author says that all, excepting Mr. Tufnell's plan of perfect rest, are attended with much danger. He thinks the treatment by iodide of potassium perfectly safe, and sure to afford relief. "It relieves the pain and all the other symptoms of aneurism more rapidly and more effectually than any other treatment, apart even from the powerful agency of the recumbent posture." He gives a score or more of histories in substantiation of this statement, and finally adds his views of the proper manner of administering the medicine, of its mode of action, and of the chances for life in cases treated by it; and we are told that there is absolutely no risk from its use. "It is almost absolutely certain to give relief and prolong life," and "No other form of treatment holds out anything like an equal prospect of relief, if not of cure."

The dose may vary in different cases from gr. v. to gr. xxx., three times a day. The latter quantity is often necessary before good results ensue, and it is apparently as well tolerated in most cases as the smaller dose, while in other cases it seems even better borne. We are advised to commence the treatment with potass. iod. ʒ ss., three times a day, conjoined with a full dose of opium or chloral at night to allay irritation and secure rest. If unpleasant results follow, the remedy should be suspended for a day or two, and then given again in large doses. In a few cases large doses cannot be employed; but it is believed that persons who experience poisonous effects from small quantities of the medicine are likely to obtain remedial effects from proportionately smaller doses of the same. Two cases are mentioned in which the remedy could not be tolerated.

The author thinks it important to saturate the system speedily with the medicine, and to maintain this condition, perhaps with slight intermissions, for several months, or at least until the symptoms are relieved. He thinks the good effects of the remedy depend, *first*, upon its sedative action on the heart, and *second*, upon a peculiar influence upon the arterial system, which not only causes contraction of the aneurismal sac (as it would of a simply dilated artery), but also induces thickening of its walls; and he does not believe the relief due to increased coagulability of the blood.

Perfect rest is an important — almost essential — element in the treatment; but there seems to be no need of restricting the diet, though fluids should be taken sparingly. Alcoholics are always injurious in these cases, excepting for temporary stimulation.

A perfect cure can be expected only in sacculated aneurisms and in those of a more or less traumatic character occurring in comparatively young individuals; though great relief and considerable prolongation of life may be confidently looked for in almost every instance where this plan of treatment is followed.

There are a few typographical errors in the book, though we notice only one of importance. On the thirty-second page the normal positions of the aortic and pulmonary sounds are said to be at "the third right and the second left costal cartilages." This might confuse the student who was not aware that these positions are at the *second* right and *third* left, etc.

We notice two or three grammatical errors, and several perplexing sentences; yet, as a whole, the work reflects credit upon its author, who has treated his subject thoroughly, and added to its standard literature some original thoughts which will render the work a valuable acquisition to the profession.

E. F. I.

A Practical Treatise on the D seases, Injuries and Malformations of the Urinary Bladder, the Prostate Gland, and the Urethra, by *Samuel D. Gross, M.D., LL.D., D.C.L.*, Professor of Surgery in the Jefferson Medical College of Philadelphia. Third edition — revised and edited by Samuel W. Gross, A.M., M.D., Surgeon to the Philadelphia Hospital. Illustrated by one hundred and seventy engravings. Philadelphia: Henry C. Lea. 1876. pp. 574.

"What shall be done unto the man whom the king delighteth to honor?" What but to say that his work is worthy of the hand that wrought it. The elder Gross has now well nigh reached the climax of the grandest success possible for an American surgeon. As an instructor, practitioner, *clinicien*, operator, biographer and author of scientific works, his eminence is to be measured by the extent of his reputation. Decorated with the doctorate of Oxford, and crowned with the highest of honors by the late International Medical Congress at Philadelphia, our author, it would seem, can hereafter add but little to the rounded completeness of his splendid professional career.

All of Dr. Gross' works are valuable, and many readers will be pleased to accord to a third edition of the present treatise the reception given to its predecessor, which has now been out of print for several years. It differs from the latter in that the chapter on the anatomy of the urinary organs has been omitted, as well as the appendix relative to the prevalence of stone in the bladder and calculus disorders in the United States. Dr. C. H. Martin, of Mobile, Alabama, has contributed for this edition, the statistics of lithotomy as performed by American surgeons, and several new engravings have been supplied by Dr. Barnes, the Surgeon General of the United States Army.

Dr. S. W. Gross, the son of the distinguished author, has been entrusted with the general work of revision, and the preface states that he has brought the work "fully up to the existing state of our knowledge." After a careful examina tion of the results of his efforts, we are prepared to endorse this statement as one which the candid reader cannot fail to accept.

Since the chapters on Tumors of the Bladder and of the Prostate Gland, are entirely written by the son, we naturally turn to them in the expectation of noting the contrast between these and other portions of the work. And yet the younger hand has produced effects which are well nigh undistinguishable in style, practical value and suggestive, critical analysis, from those to which the medical public has been long accustomed.

In alluding, however, to the histological structure of the hypertrophied prostate, it is here stated that the anomalous condition is due to hyperplasia of the muscular and fibrous elements at the expense of the glandular structures "which disappear in part or entirely." How could there possibly be, after this partial or complete disappearance of the glandular structures, that "free discharge of prostatic fluid," which is enumerated among the symptoms of advanced and severe cases? It is more than probable that the histological status of the prostate, when hypertrophied, is just the reverse of that described. The fibrous elements are rarely affected, while the proliferation of muscular and glandular elements is in active progression. Iverson declares that this hypertrophy is due to "a myomatous, or myoadenomatous hyperplasia," while, according to Rindfleisch, there is "a fibro-muscular overgrowth of the peritubular stroma of single segments of the gland, with a coincident *elongation* and *multiplication* of the tubuli themselves."

There is one caution which the professional reader should observe in the consideration of this chapter, and that is not to experiment too fearlessly with the plan of treatment recommended by Professor Heine of Innsbruck. This method consists in injecting the gland through the anterior wall of the rectum, with solutions of iodine, by the aid of a Pravaz syringe. Heine's solution is composed of two drachms of the iodide of potassium, two ounces of tincture of iodine and *two* ounces of water (not *six* as stated in the text). The younger author is of opinion that "this plan of treatment deserves more extended trial," while he admits that there is risk of suppuration. Our advice on this point is that lately proffered by the *London Punch* to young men contemplating matrimony, "Don't!"

Already Prof. Dittel of the General Hospital at Vienna—a man of unsurpassed experience in diseases of the prostate gland—has had a fatal case resulting from two injections merely, each of four drops of Heine's solution. When last heard from, Prof. Dittel did not propose to experiment with it again, as he had combatted without success, in this particular case, peri-prostatitis, peri-urethritis, peri-urethritic abscess, peri-proctitis, cystitis, pyelitis and nephritis!

Of the work as a whole, we need but say that it is fully equal in value to the other treatises by its eminent author, in order to pronounce its highest encomium.

